# Giant Type IV hiatal hernia with intrathoracic migration of abdominal viscera and atypical clinical presentation: a case report

**DOI:** 10.1093/jscr/rjag816

**Published:** 2026-09-16

**Authors:** Ilkhom B Khayitov, Akhmadjon B Babajonov, Izzatulla V Khakimov, Diana I Yugay

**Affiliations:** Department of Surgical Diseases in Family Medicine, Tashkent State Medical University, Farabi Street 2, Almazar District, Tashkent, 100109, Uzbekistan; Department of Surgical Diseases in Family Medicine, Tashkent State Medical University, Farabi Street 2, Almazar District, Tashkent, 100109, Uzbekistan; Department of Surgical Diseases in Family Medicine, Tashkent State Medical University, Farabi Street 2, Almazar District, Tashkent, 100109, Uzbekistan; Department of Surgical Diseases in Family Medicine, Tashkent State Medical University, Farabi Street 2, Almazar District, Tashkent, 100109, Uzbekistan

**Keywords:** paraesophageal hernia, gastric volvulus, laparoscopic repair, mesh hiatoplasty, atypical presentation, case report

## Abstract

The Type IV paraesophageal hernia is the rarest and most treacherous hiatal hernia: herniated viscera compressing the chest cavity can imitate cardiopulmonary symptoms convincingly enough to derail the diagnostic workup. We describe a 57-year-old man in whom a Type IV hiatal hernia, which found incidentally 25 years earlier, had enlarged into a giant 15 × 12 cm defect with a 180-degree organoaxial gastric volvulus. He presented not with reflux but with orthopnoea, nocturnal cough, postprandial palpitations, and muffled heart sounds; only after a normal cardiac workup did computed tomography reveal stomach, colon, and small bowel within the thorax. Laparoscopic reduction, sac excision, cruroplasty, Nissen fundoplication, and mesh hiatoplasty resolved every symptom, with an intact repair confirmed at three years. This case captures the natural history of a neglected hernia and argues for early cross-sectional imaging when cardiopulmonary symptoms outlast a normal cardiac and pulmonary evaluation.

## Introduction

Type IV hiatal hernias, where stomach and other viscera sit in the chest, make up under 5% of cases yet carry the highest risk of obstruction, volvulus, and strangulation [1]. Some of the patients report reflux or dysphagia. When a large hernia compresses the mediastinum, the presenting complaint may be breathlessness or palpitations, and the workup may start in a cardiology or respiratory clinic rather than a surgical department [1, 2]. Diagnosis is then delayed, and where elective surgery is paid for out of pocket, that delay stretches further still.

What makes this case worth reporting is not the operation but its history. A hernia found incidentally a quarter of a century ago grew, unwatched, into a giant Type IV defect with organoaxial volvulus and declared itself through the heart rather than the gut. We describe the presentation, the laparoscopic mesh-reinforced repair, and a documented 3-year result rarely reported for hernias of this size.

## Case report

A 57-year-old man was referred with several months of progressive orthopnoea, chronic cough, postprandial palpitations, and retrosternal discomfort. A small hiatal hernia had been diagnosed endoscopically 25 years earlier; he had not been reviewed since. He was a lifelong non-smoker with no previous abdominal surgery, no cardiorespiratory history, and no regular medications. Body mass index was 27. Family history was unremarkable.

**Figure 1:**
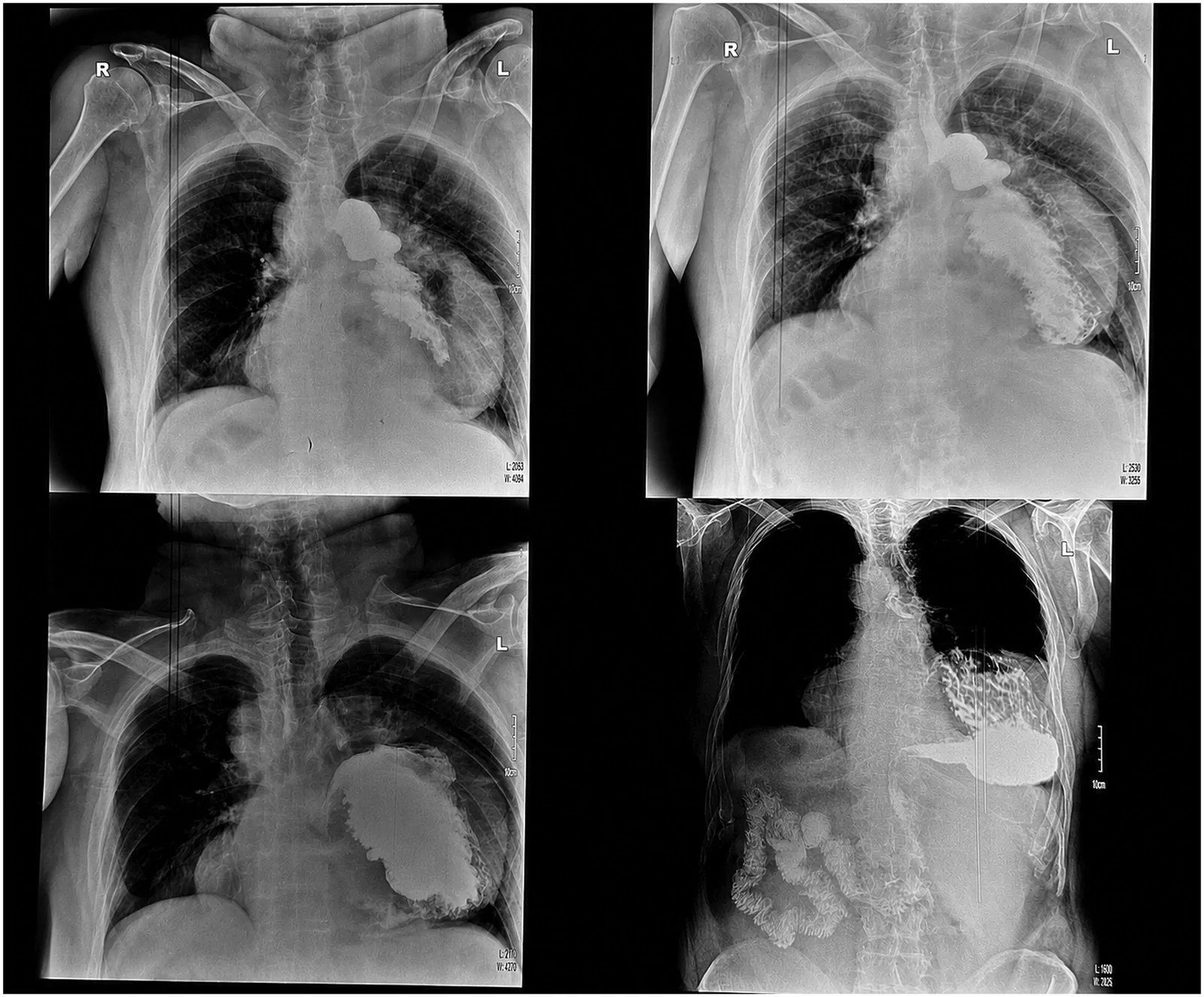
Barium study. Contrast-filled stomach displaced into the thorax with the mucosal pattern showing organoaxial rotation.

**Figure 2:**
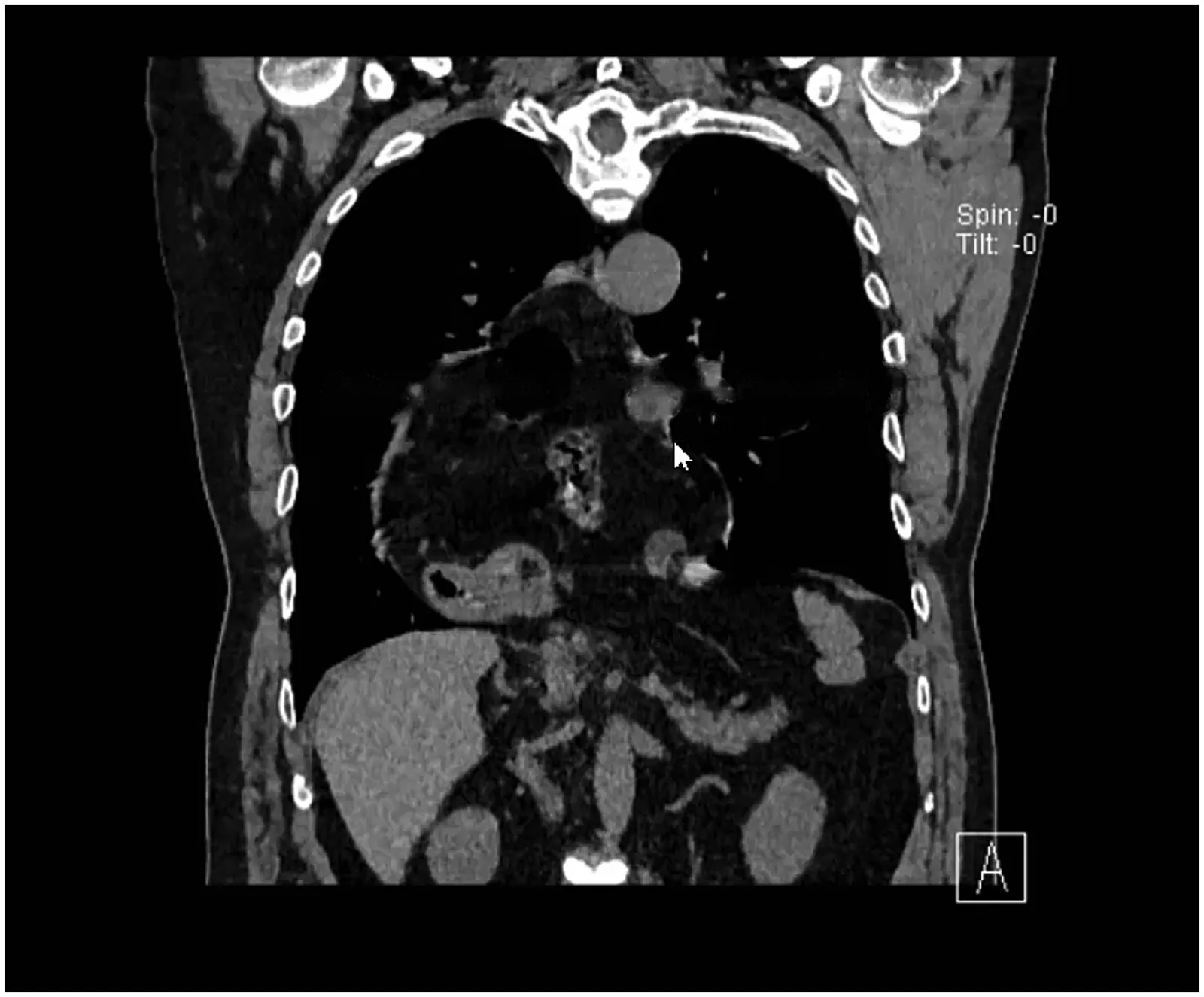
Multi-slice CT (coronal). Giant Type IV paraesophageal hernia (20 × 23 cm hernia sac) with stomach, colon, and small bowel within the thorax.

He was tachycardic (100–110 bpm) with a blood pressure of 140/90 mmHg, pale, with muffled heart sounds and reduced breath sounds at the left base. The abdomen was soft. Haemoglobin was 126 g/l; renal and liver function, coagulation, and remaining tests were normal. Electrocardiography showed sinus tachycardia, voltage criteria for left ventricular hypertrophy, and lateral T-wave flattening, without ischemic change. Echocardiography confirmed left ventricular hypertrophy with preserved systolic function (ejection fraction improved from 56% before to 64% after preoperative preparation) and no valvular or pericardial abnormality. Chest radiography showed an elevated left hemidiaphragm with the lung base displaced to the third–fourth rib level. Barium study demonstrated a grade 3–4 hiatal hernia, and endoscopy showed gastric torsion with Savary–Miller grade III oesophagitis. Multi-slice computed tomography (CT) defined a hiatal orifice with the size 15 × 12 cm Type IV paraesophageal hernia containing stomach, colon, and small bowel within the thorax, complicated by a 180° organoaxial gastric volvulus and dense mediastinal adhesions (Figs 1 and 2).

**Figure 3:**
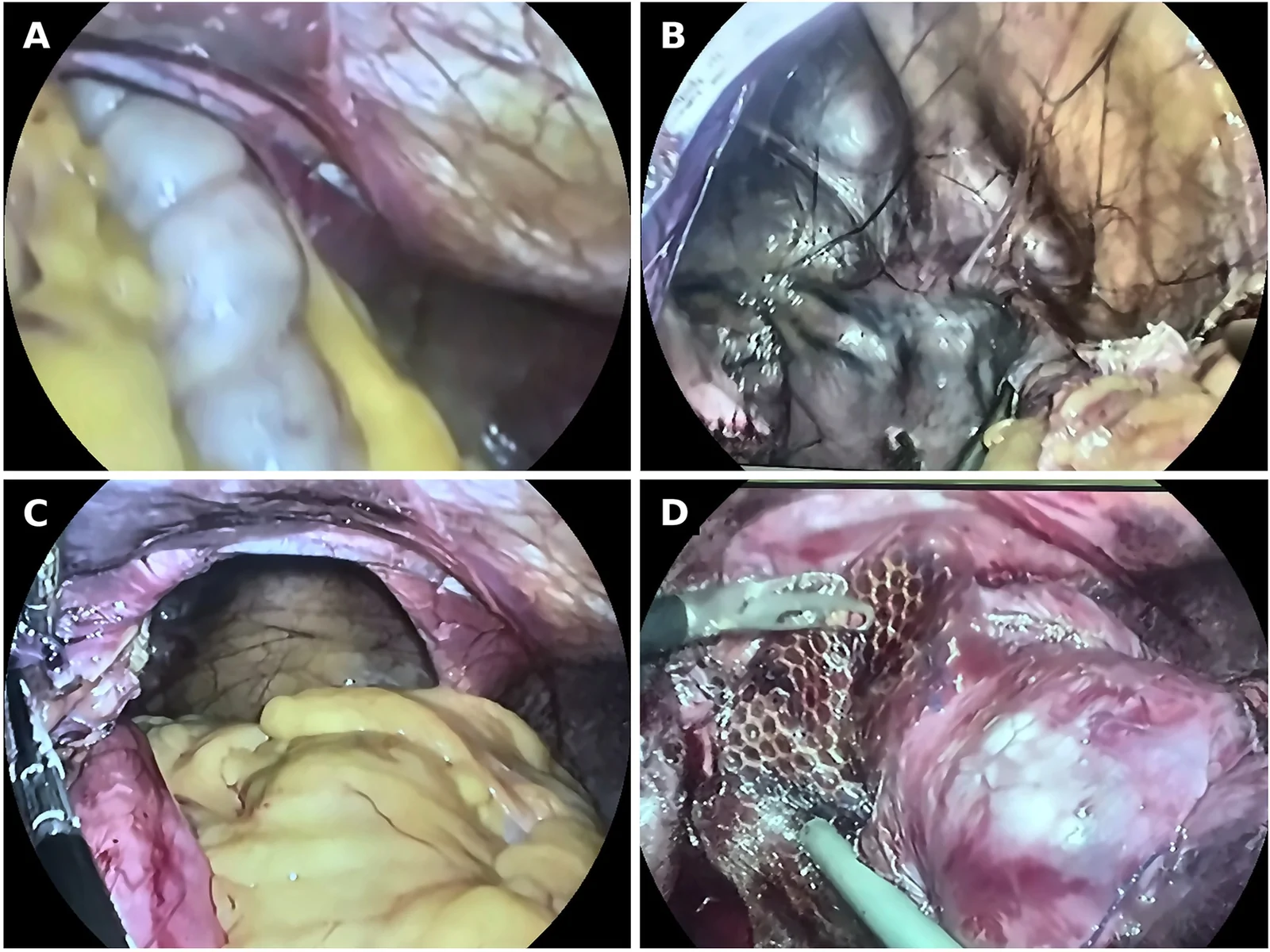
Intraoperative laparoscopic views. (A) Reduction of the colon from the thorax into the abdomen. (B) Stomach herniated within the sac in the posterior mediastinum before reduction. (C) The hiatal defect after complete reduction of the herniated viscera. (D) Coated polypropylene mesh positioned over the crural repair and fixed with titanium tackers.

After cardiology and anaesthetic assessment, laparoscopic repair was performed under general anaesthesia through five ports by a surgeon with 10 years of experience in laparoscopic foregut surgery, with antibiotic prophylaxis and thromboprophylaxis. Adhesiolysis freed the herniated viscera, which were reduced with technical difficulties, and the stomach was detorsed. The hernia sac was dissected from the mediastinum and excised. The oesophagus was mobilized until at least 3 cm of tension-free intra-abdominal length was achieved. The crura were approximated with three posterior and one anterior interrupted non-absorbable sutures. A 360° Nissen fundoplication was fashioned over a 50 Fr bougie, and coated polypropylene mesh was fixed over the repair with titanium tackers, kept away from the oesophagus and pericardium (Fig. 3A–D). A closed suction drain was left subphrenically. Operating time was 127 min with minimal blood loss.

Recovery was uncomplicated. Clear fluids were started on Day 1 and a soft diet on Day 3; the drain was removed on Day 2 and he was discharged on Day 4. Pantoprazole 40 mg daily was given for 1 month, then intermittently. He was reviewed at 1, 6, 12, 24, and 36 months. Endoscopy at 24 months showed a normal gastro-oesophageal junction with an intact wrap and no recurrence or oesophagitis. At 36 months, he was free of dysphagia, reflux, and cardiorespiratory symptoms.

## Discussion

The instructive feature here is a mechanical foregut catastrophe presenting as cardiopulmonary disease. Orthopnoea, cough, postprandial palpitations, muffled heart sounds, and persistent sinus tachycardia directed evaluation towards the heart, and the diagnosis emerged only on cross-sectional imaging. Such presentations are well recognized and rank among the principal reasons giant paraesophageal hernias are identified late, as mediastinal compression generates cardiorespiratory symptoms mimicking primary cardiac and respiratory pathology [1–3]. The implication is specific: when cardiac evaluation is unremarkable and symptoms are reproducibly postprandial, early CT of the thorax and abdomen is the most informative investigation and should not be deferred.

The second noteworthy feature is the protracted natural history. When first identified 25 years earlier the hernia was small and endoscopically unremarkable, yet never subsequently surveilled. The absence of follow-up reflected structural barriers to care rather than clinical oversight: elective investigation and surgery are not publicly funded here, and the resulting out-of-pocket cost, compounded by limited access, deterred re-evaluation. Over the ensuing decades an eminently repairable defect progressed to a 15 × 12 cm Type IV hernia with a 180° organoaxial volvulus and dense adhesions—a trajectory reflecting interrupted surveillance rather than the intrinsic behaviour of the disease.

Despite the size of the defect and dense adhesions, a laparoscopic approach remained feasible and conversion was avoided. For defects exceeding 10 cm, primary suture cruroplasty carries an unacceptably high recurrence rate, and prosthetic reinforcement reduces this risk [4–6]. The principal concern with synthetic mesh at the hiatus is erosion; the prosthesis was therefore secured with titanium tackers positioned away from the oesophagus and pericardium, though such a construct mandates sustained surveillance [7]. Reported recurrence rate after mesh repair of large hernias ranges is 12.4% [5]; here, endoscopy at 24 months and review at 36 months confirmed a durable, symptom-free result. A single case cannot establish comparative efficacy, but it demonstrates durability in a technically demanding setting.
